# Age-Related Gene Expression in the Frontal Cortex Suggests Synaptic Function Changes in Specific Inhibitory Neuron Subtypes

**DOI:** 10.3389/fnagi.2017.00162

**Published:** 2017-05-29

**Authors:** Leon French, TianZhou Ma, Hyunjung Oh, George C. Tseng, Etienne Sibille

**Affiliations:** ^1^Neurobiology of Depression and Aging Lab, Centre for Addiction and Mental Health, Campbell Family Mental Health Research InstituteToronto, ON, Canada; ^2^Department of Psychiatry, University of TorontoToronto, ON, Canada; ^3^Institute of Medical Science, University of TorontoToronto, ON, Canada; ^4^Department of Biostatistics, University of PittsburghPittsburgh, PA, United States; ^5^Department of Pharmacology and Toxicology, University of TorontoToronto, ON, Canada

**Keywords:** gene expression, cell-type specific, transcriptome, aging, neuroinformatics, synapse, cortex

## Abstract

Genome-wide expression profiling of the human brain has revealed genes that are differentially expressed across the lifespan. Characterizing these genes adds to our understanding of both normal functions and pathological conditions. Additionally, the specific cell-types that contribute to the motor, sensory and cognitive declines during aging are unclear. Here we test if age-related genes show higher expression in specific neural cell types. Our study leverages data from two sources of murine single-cell expression data and two sources of age-associations from large gene expression studies of postmortem human brain. We used nonparametric gene set analysis to test for age-related enrichment of genes associated with specific cell-types; we also restricted our analyses to specific gene ontology groups. Our analyses focused on a primary pair of single-cell expression data from the mouse visual cortex and age-related human post-mortem gene expression information from the orbitofrontal cortex. Additional pairings that used data from the hippocampus, prefrontal cortex, somatosensory cortex and blood were used to validate and test specificity of our findings. We found robust age-related up-regulation of genes that are highly expressed in oligodendrocytes and astrocytes, while genes highly expressed in layer 2/3 glutamatergic neurons were down-regulated across age. Genes not specific to any neural cell type were also down-regulated, possibly due to the bulk tissue source of the age-related genes. A gene ontology-driven dissection of the cell-type enriched genes highlighted the strong down-regulation of genes involved in synaptic transmission and cell-cell signaling in the Somatostatin (Sst) neuron subtype that expresses the cyclin dependent kinase 6 (Cdk6) and in the vasoactive intestinal peptide (Vip) neuron subtype expressing myosin binding protein C, slow type (Mybpc1). These findings provide new insights into cell specific susceptibility to normal aging, and suggest age-related synaptic changes in specific inhibitory neuron subtypes.

## Introduction

Aging is a key factor for several neuropsychiatric disorders and for most neurodegenerative diseases with onset typically occurring late in life and with worsening of symptoms over time (Glorioso and Sibille, 2011). Understanding the effects of aging at the cellular level can provide possible treatment insights into many neuropsychiatric, neurodegenerative disorders. It also has the potential to explain age related declines in many motor, sensory and cognitive abilities (Ianov et al., 2016). Genome-wide expression profiling of postmortem brain tissue has found hundreds of age-related genes that are differentially expressed in an age dependent manner (Lu et al., 2004; Erraji-Benchekroun et al., 2005; Colantuoni et al., 2011; Berchtold et al., 2013; Kumar et al., 2013). Age up-regulated genes are enriched for neuroinflammatory (Bordner et al., 2011) and overall glial (Erraji-Benchekroun et al., 2005) functions. In contrast, age down-regulated genes are most commonly involved in synaptic function and cell-cell signaling (Primiani et al., 2014). These associations suggest cell-type specific changes in glial and neuronal cells (Erraji-Benchekroun et al., 2005). More directly, Loerch et al. (2008) tested age associations of genes enriched in three main cell classes, where they found that neuronal genes were down-regulated and astrocyte and oligodendrocyte enriched genes were up-regulated with age. Most recently, cell-type composition of 10 primary cell-types were estimated from collated lists of cell-type specific genes (Hagenauer et al., 2016). In prefrontal samples, the neuronal content estimates were negatively correlated with age with no effect for the remaining nine cell-types. However, to our knowledge, genes that mark the wide diversity of neural cell types have not been examined in the context of aging.

Advances in techniques for single cell isolation and RNA sequencing have revealed transcriptomic profiles of individual cells. Using these methods, two large studies of mouse cortex have assayed and clustered individual cells into over 40 transcriptomic cell-type classes (Zeisel et al., 2015; Tasic et al., 2016). These studies provide extended sets of enriched and marker genes that can help determine which cells are contributing to signals found in studies of bulk brain tissue (Xu et al., 2014; Mancarci et al., 2016; Skene and Grant, 2016). This should enable a finer dissection of age-related changes in gene expression.

**Figure 1:**
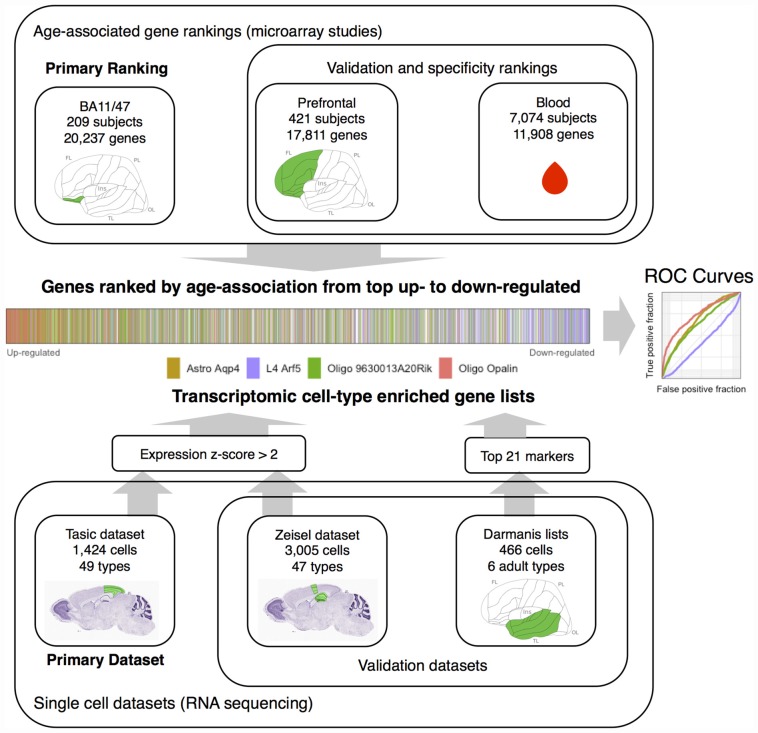
**Workflow overview of the study**. Gene rankings from aging studies are presented at top. Single cell datasets are presented in the lower portion. The middle visualizes the joining of the rankings and single cell datasets by marking transcriptomic cell-type enriched genes within an age-associated gene ranking. This can also be represented with an Reciever operating characteristic (ROC) curve (middle right). The Nissl stained mouse brain sections and the template diagram of the human cortex mark the source brain regions in green (images from the Allen Institute for Brain Science atlases, Lein et al., 2006; Ding et al., 2016). Abbreviations: FL, frontal lobe; OL, occipital lobe; Ins, insula; TL, temporal lobe; PL, parietal lobe, Astro, astrocyte; L4, layer 4, Oligo, oligodendrocyte.

In our current study, we used genes with cell-type specific expression to characterize age-related differences in expression obtained from bulk tissue, i.e., combined gray matter samples comprised of all six cortical layers. We used data obtained from both mouse and human cerebral cortex samples. We integrated two studies of brain aging with two cell-type specific studies in mouse to provide a robust estimate of cell-type associated changes in the aging brain (Figure 1). A third transcriptomic study of age-related genes from blood and a third cell-type study of human neural cells provided supplementary data. Furthermore, within cell-type enriched gene sets, we tested whether genes annotated to specific functions or processes were up- or down-regulated across age.

## Materials and Methods

### Age Associated Gene Rankings

Three gene expression studies of aging were used to rank genes from the most significant up- to down-regulated with age. Two studies examined expression in the frontal cortex and a third study used whole blood samples, allowing testing of neural specificity.

#### BA11/47 Ranking

This postmortem dataset of orbitofrontal cortex expression profiles has been described previously (Seney et al., 2013; Lin et al., 2015; Chen et al., 2016). This data is available from the Gene Expression Omnibus repository under the accession number GSE71620. Briefly, samples were obtained from Broadmann areas 11 and 47 for 178 Caucasian and 31 African-American individuals without any DSM-IV diagnosis for cognitive or neurodegenerative disorders. Age of the 209 subjects ranged from 16 years to 91 years old. Gene expression was profiled with the Affymetrix GeneChip 1.1 ST according to manufacturer’s protocol and Robust Multi-array averaging (RMA) was used for normalization (Irizarry et al., 2003). For each gene, association with age was determined by a random intercept model with covariates for brain pH (mean 6.7), post-mortem interval (mean 17.2 h) and sex (79% male) in each Broadmann area separately. The Adaptively Weighted (AW) fisher method was used to combine *p*-values and direction of effect across the two regions (Li and Tseng, 2011). The final ranking of 20,237 genes was ordered by AW Fisher *p*-value and direction (from the up-regulated gene with the lowest *p*-value to the down-regulated gene with lowest *p*-value). We henceforth refer to this ranking as the BA11/47 ranking.

#### Prefrontal Ranking

A cross-laboratory meta-analysis of expression profiling data from normal postmortem cortex provided a second ranking of age associations (Mistry and Pavlidis, 2010). This study combined expression data from 11 expression studies that profiled 421 normal subjects. Age of the subjects ranged from 19 years to 106 years old (57% male). The frontal cortex was the assayed region for all but one of the included datasets. The dorsolateral prefrontal cortex was used in four of the studies. RMA normalization was used to normalize the majority of datasets used. Association with age for each gene were obtained from Supplementary Table 10. Because this analysis was split by direction of change, for each gene we choose the result with the lowest *p*-value. The result is an up- to down- regulated ranking as above for 17,811 genes. We henceforth refer to this ranking as the prefrontal ranking.

#### Blood Ranking

A large gene expression meta-analysis provided a third ranking based on age associations in whole-blood (Peters et al., 2015). This discovery analysis combined six studies (using either Illumina or Affymetrix arrays), providing peripheral blood expression profiles for 7074 individuals of European ancestry. *P*-values and direction of age-associated expression for the 11,908 genes tested in the discovery stage of the Peters et al. (2015) study were obtained from column “P” in Supplementary data file 1.

### Cell-Type Enriched Genes

#### Tasic Dataset

Single-cell expression profiles from the mouse primary visual cortex are our main source of cell-type enriched genes (Tasic et al., 2016). This RNA sequencing dataset contains information for 1679 single cells that have been clustered into 49 named groups. Groups were named using known cell-type marker genes, source dissection cortical layer, *in situ* hybridization images, and dissections of specific Cre lines. We used the 1424 “core” cells that were reliably classified into a single cell-type cluster by Tasic et al. (2016) Two additional single cells in the (“Smooth muscle cells marked by Myl9, SMC Myl9”) cluster that had the major cell type class listed as unknown were removed, leaving 1422 cells. Expression data was obtained from the Allen Cellular Taxonomy Case study website^1^. Genes with zero reads for all cells were removed (2677). Gene symbols were updated with the mygene R package (Xin et al., 2016). For the remaining 21,380 genes, we log transformed the provided RPKM expression values plus one. For each gene, these log scaled values were standardized across all cells. Cells were then grouped by provided 49 transcriptomic types and the average standardized expression value was calculated for each gene. Genes with average standardized expression levels higher than two standard deviations in a given transcriptomic cell type were considered cell-type enriched. An additional cell type nonspecific list of genes with stable expression across the cell types was created by selecting genes with an absolute average standardized expression less than two for all 49 transcriptomic cell types.

#### Zeisel Dataset

A secondary source of expression data assayed cells from the mouse hippocampus and cortex (Zeisel et al., 2015). Expression data (number of molecules per cell) was obtained from the Linnarson lab website^2^). This dataset assayed 3005 cells from the somatosensory (S1) cortex and hippocampus. We used the provided BackSPIN clustering that marked cells as one of seven major classes (“level1class” in data file) and 47 cell subclasses. The seven major classes are named: interneurons, pyramidal SS, pyramidal CA1, oligodendrocytes, microglia, endothelial-mural and astrocytes_ependymal. Unlike the Tasic dataset, most of the 47 subclasses have anonymous names like “Oligo6” but do include some names based on known markers, layer or region information. Genes with average standardized expression levels higher than two standard deviations in a specific cell class and a nonspecific gene list was created using the same method applied to the Tasic dataset.

### Mapping of Mouse to Human Gene Symbols

The cell-type associated gene lists from the mouse single cell datasets were converted to human gene symbols using the Homologene database (O’Leary et al., 2016) and homologene R package by Ogan Mancarci^3^. Analyses of the age associated rankings was restricted to human genes that had a homologene mapping from the genes assayed in the mouse single cell datasets.

### Enrichment Tests

The area under the receiver operating curve (AUROC) statistic was used to measure enrichment for a cell-type enriched gene set in an age associated gene ranking. This value is equal to the probability that a cell-type enriched gene will rank higher in an age-associated ranking than a gene not enriched for the given transcriptomic cell-type. We used fast AUROC methods from EGAD (Ballouz et al., 2017). The provided cell-type identities or subclasses in the Tasic and Zeisel datasets were permuted to determine the empirical *p*-values of the AUROCs. False discovery rate was used to correct for multiple tests. This empirical procedure was chosen because age associated cell-type AUROCs reached 0.55 instead of the expected chance performance (0.5) for randomly permuted Tasic and Zeisel datasets. These AUROCs from the permuted cell-type data correspond to Mann-Whitney (Wilcoxon) test *P*-values < 0.005 (AUROC = 0.55). This shift from 0.5 is due to many non-specific genes being consistently down-regulated (more details are provided in the “Results” Section). In the empirical procedure, for a given cell-type transcriptomic class, AUROC values are calculated for 10,000 random assignments of transcriptomic cell-type. The proportion of permuted shuffles with a larger or smaller AUROC value in comparison to the real cell type classification is the *p*-value (relative to the average empirical AUROC). Due to the finite number of permutations the floor of this *p*-value is set to 1/10,000 or 0.0001 for the cases of no permuted shuffles with a larger or smaller AUROC values. The false discovery rate *q*-value is computed by counting the proportion of random assignments that have at least one cell-type with an equal or lower *p*-value for any transcriptomic cell-type class.

### Supplemental Gene Sets

In addition to the mouse datasets above, human gene sets were used to examine cell-type enrichment in the age rankings. From the first single cell transcriptome analysis of healthy human adult cortical samples, we obtained six lists of the top 21 most enriched genes in six transcriptomic determined cell-types (via clustering, Supplementary Table S3; Darmanis et al., 2015). This dataset profiled expression in 466 human cortical cells that were obtained from four embryonic samples and eight adult samples. Selected cell clusters excluded gene sets corresponding to clusters of mixed and fetal cell types. Top 21 lists identified as astrocytes, neurons, oligodendrocytes, oligodendrocyte precursors, microglia and endothelial cell clusters remained. The number of genes tested in each ranking varies because not all of these 21 genes were assayed in the age-associated datasets. Bonferroni multiple test correction was applied to correct for the six tests in a given ranking. Separately, a list of housekeeping genes that are expected to maintain constant expression levels in all cells was also used (Eisenberg and Levanon, 2013). RNA-seq data from 16 human tissues (including brain) was used to create this list of 3804 genes.

### Gene Ontology Analysis

Gene ontology gene groups were obtained using the GO.db and org.Hs.eg.db packages in R (Carlson, 2016a,b). The annotations were recent, with a GO source version date of March 14,2016. GO annotations from all three domains were used (cellular component, molecular function and biological process). Cell-type enriched human gene sets were ranked using the age associations. Within these rankings, we calculated the AUROCs for GO groups that were over 10 and less than 200 genes in size after intersection with the specific cell-type list. Mann-Whitney (Wilcoxon) test *p*-values were calculated and corrected for the many cell-type by GO group tests (FDR method).

### Availability

Scripts and data files for the analyses are available online at https://github.com/leonfrench/CellTypesAging.

## Results

### Comparison of Age-Associated Gene Rankings

We first compared the three age-associated rankings. Across the 9032 genes that were assayed in all three studies, the highest correlation was between the two cortical datasets (Spearman’s rho = 0.597, *p* < 0.0001). The Prefrontal ranking was weakly correlated with the Blood age associations (rho = 0.06, *p* < 0.0001) but the orbitofrontal BA11/47 ranking was not (rho = 0.007, *p* > 0.5).

In this article, we focus on the BA11/47 ranking and Tasic cell-type dataset due to its use of targeted transgenic Cre lines. Selection and conversion of cell-type enriched lists from the Tasic single cell dataset resulted in 17,590 mouse genes that are enriched in at least one of the 49 cell types. The number of enriched genes per cell type ranged from 221 to 1500. In contrast, 3781 genes did not show clear enrichment for a specific cell type. These gene lists are reduced after conversion to human genes and intersected with a specific age associated ranking.

### Cell-Type Enriched Gene Set Tests

**Figure 2:**
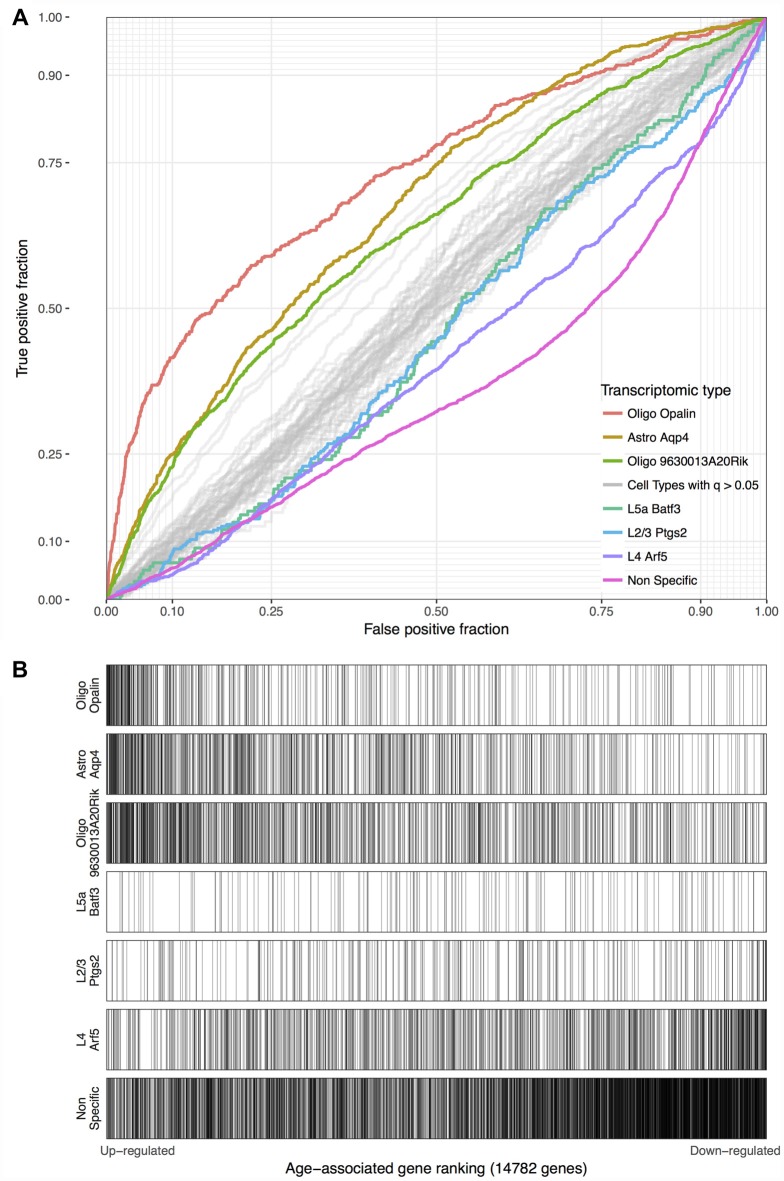
**(A)** ROC curves for the seven significant cell-type enriched gene sets from the Tasic dataset, projected on the BA11/47 age ranking. The curves show the proportion of transcriptomic-type enriched genes that overlap (*y*-axis, true positive fraction) in varying lengths of the age-associated gene ranking (approximated by the *x*-axis, false positive fraction). Colored lines mark genes enriched in transcriptomic cell types that were age-associated after correction and light gray lines mark the remaining cell types that were not. **(B)** Distributions of the seven significant gene sets across the age-associated ranking with each transcriptomic cell-type enriched gene representing a single black line. Gradient bars that begin as darker shades and move to white mark age up-regulated enrichment, with the opposite pattern for down-regulation. The gene sets are arranged from most up-regulated (Oligo Opalin) to most down-regulated (Non-specific genes).

Using the Tasic genes lists and BA11/47 ranking, we next tested if specific cell type linked genes were over- or under-expressed with age (Figure 2 and Table 1). Genes with enriched expression in Oligodendrocytes marked by the Opalin or 9630013A20Rik genes, and Astrocytes were upregulated with age in the BA11/47 samples (AUROC > 0.63, empirical *q* < 0.05). In the context of neurons, all but one of the 42 neuron-type gene lists were down-regulated with age but only three were significant after correction. Specifically, genes enriched in glutamatergic layer 2/3 (marked by the Ptgs2 gene), layer 4 (Arf5) and layer 5 (Batf3) neurons showed lower expression with age (AUROC < 0.47, *q* < 0.05). Genes lacking clear enrichment for a specific cell type were also expressed at lower levels in older samples (AUROC = 0.37, *q* < 0.02). This enrichment for age-associated down-regulation of non-specific genes was observed with random assignments of cell-type (Table 1, Permuted AUROC = 0.414). Because a gene cannot be both non-specific and cell-type enriched, this down-regulation of broadly expressed genes results in empirical AUROCs above 0.5 for genes enriched in any subset of cells (Table 1, mean Permuted AUROC = 0.534, max = 0.56). Across the 49 cell-types, the number of cells per cell-type was negatively correlated with age-associated *p*-values (rho = −0.58, *p* < 0.0001). This maybe due to rare cell-types contributing less signal in the aging studies of bulk tissue. In contrast, the number of enriched genes per cell-type was not correlated with placement in the BA11/47 ranking (rho = 0.07, *p* > 0.5).

**Table 1:** **Age associations of transcriptomic cell-type enriched gene sets using the Tasic dataset and BA11/47 ranking (see abbreviation list)**.

Transcriptomic type	Cell count	Gene count	Permuted AUROC	AUROC	*p*	*q*
Oligo Opalin	30	448	0.544	0.730	0.0001	0.005
L2/3 Ptgs2	92	316	0.560	0.455	0.0001	0.005
Astro Aqp4	43	830	0.553	0.673	0.0003	0.0146
Non-specific	NA	3257	0.414	0.368	0.0004	0.0195
L4 Arf5	31	1097	0.545	0.411	0.0005	0.0243
L5a Batf3	57	158	0.558	0.462	0.0005	0.0243
Oligo 9630013A20Rik	7	1089	0.499	0.630	0.001	0.048
SMC Myl9	11	1095	0.511	0.639	0.0014	0.0669
Sst Cbln4	66	156	0.560	0.490	0.0022	0.1031
L6a Sla	53	168	0.557	0.484	0.0051	0.2282
Vip Parm1	38	127	0.550	0.466	0.0078	0.326
Vip Gpc3	45	160	0.554	0.475	0.0081	0.3359
L5a Hsd11b1	42	330	0.552	0.475	0.0083	0.3434
Endo Xdh	14	738	0.520	0.625	0.009	0.3678
L4 Scnn1a	64	271	0.559	0.521	0.0793	0.9845
Vip Chat	46	187	0.555	0.511	0.0801	0.9851
L6a Mgp	37	207	0.549	0.498	0.0824	0.9869
OPC Pdgfra	22	828	0.534	0.594	0.1137	0.9979
Ndnf Cxcl14	30	153	0.544	0.497	0.1467	0.9996
Sst Myh8	38	181	0.550	0.509	0.1513	0.9996
Pvalb Wt1	46	431	0.555	0.519	0.1513	0.9996
Sst Chodl	41	536	0.552	0.514	0.16	0.9998
Pvalb Tacr3	63	393	0.559	0.530	0.1771	0.9999
Vip Mybpc1	24	187	0.537	0.495	0.2407	1
L5b Cdh13	30	396	0.544	0.509	0.2695	1
L5b Tph2	25	717	0.538	0.502	0.3059	1
Sst Th	16	297	0.524	0.564	0.3282	1
L4 Ctxn3	55	123	0.558	0.536	0.34	1
L6b Rgs12	13	745	0.517	0.477	0.3426	1
Micro Ctss	22	1030	0.534	0.565	0.3965	1
L6b Serpinb11	16	488	0.524	0.492	0.4305	1
L5a Tcerg1l	20	380	0.531	0.506	0.5127	1
Pvalb Rspo2	21	567	0.533	0.556	0.5331	1
L6a Car12	14	508	0.519	0.496	0.5719	1
Vip Sncg	13	397	0.517	0.494	0.5822	1
L5a Pde1c	12	391	0.514	0.491	0.5926	1
Igtp	10	439	0.510	0.533	0.6176	1
Sst Tacstd2	12	605	0.515	0.495	0.6566	1
Sst Cdk6	14	389	0.519	0.501	0.6635	1
Smad3	12	679	0.515	0.500	0.7292	1
Ndnf Car4	24	246	0.537	0.528	0.7871	1
Pvalb Cpne5	14	315	0.519	0.528	0.8163	1
L5b Chrna6	8	824	0.504	0.493	0.8324	1
Pvalb Gpx3	54	131	0.557	0.553	0.8366	1
Sncg	9	703	0.506	0.515	0.8503	1
L5 Ucma	12	611	0.515	0.523	0.8513	1
Pvalb Obox3	16	968	0.524	0.519	0.904	1
L6a Syt17	12	594	0.515	0.519	0.9227	1
L2 Ngb	16	536	0.524	0.527	0.9444	1
Pvalb Tpbg	12	315	0.514	0.512	0.9497	1

### Validation in Additional Age-Associated Gene Rankings

We next used the Prefrontal and Blood age-associated gene rankings to seek an independent validation of our finding by testing the tissue specificity of the BA11/47 findings (Table 2). We note that the number of human genes in these rankings varied (BA11/47: 14,782 genes, Prefrontal: 11,661, Blood: 10,585 (counts after homologene mapping)) and that the origin of brain cortical tissue also varied across the datasets. Nonetheless, we demonstrated that the seven significant cell-type gene lists identified in the BA11/47 dataset were also enriched in the Prefrontal ranking (empirical *p* < 0.04 and matching directions for all types). Also, the AUROC values correlated across the seven gene lists with only the Astrocyte cells marked by Aqp4 (Astro Aqp4) and Oligo Opalin types switching places for the highest AUROC value between the BA11/47 and Prefrontal results (Pearson *r* = 0.94). In the Blood ranking, only genes that lacked cell-type enrichment mirrored the findings in the brain datasets with lower expression in older blood samples (*p* < 0.0005), consistent with the fundamental differences in cell type compositions between the two tissues types.

**Table 2:** **Cell-type enriched gene set enrichment across three age-associated gene rankings**.

Ranking:	BA11/47			Prefrontal		Blood	
Transcriptomic type	AUROC	*p*	*q*	AUROC	*p*	AUROC	*p*
Oligo Opalin	0.73	0.0001	0.005	0.65	0.035	0.58	0.42
L2/3 Ptgs2	0.46	0.0001	0.005	0.48	0.0074	0.58	0.35
Astro Aqp4	0.67	0.0003	0.015	0.69	0.0009	0.58	0.87
Non-specific	0.37	0.0004	0.02	0.31	0.0001	0.41	0.0001
L4 Arf5	0.41	0.0005	0.024	0.45	0.0062	0.54	0.63
L5a Batf3	0.46	0.0005	0.024	0.49	0.022	0.56	0.34
Oligo 9630013A20Rik	0.63	0.001	0.048	0.63	0.013	0.51	0.49

### Confirmation with Alternate Sources of Single Cell Expression Information

Next, to control for the source of single cell expression data, we tested our results in the Zeisel dataset. Cell-type enriched lists from the dataset contain 18,658 mouse genes that are enriched in at least one of the 47 cell types. In contrast, 1314 genes show no enrichment for a specific cell type. Based on the given cell-type labels, we mapped the six significant gene groups found in the Tasic dataset to 12 cell-types in the Zeisel dataset. This expansion is primarily due to six classes of oligodendrocytes in the Zeisel dataset (two in the Tasic set). Mapping between these classes was guided by the expression levels of the Opalin and 9630013A20Rik marker genes. Using the Zeisel dataset, the direction of effect for the 24 relationships (12 mapped cell-types by two brain based age rankings) matched the Tasic based findings (Table 3). However, age-associated down-regulation of layer 5a pyramidal neuron (S1PyrL5a) genes tested with the BA11/47 or Prefrontal rankings did not reach significance. Down-regulation of layer 4 pyramidal neuron (S1PyrL4) genes and up-regulation of one oligodendrocyte class (Oligo1) tested with the Prefrontal ranking failed to reach significance but have similar AUC scores and directions of effect. Like the Tasic findings, AUROCs for the 12 Zeisel based gene lists are correlated between the BA11/47 and Prefrontal results (Pearson *r* = 0.94). No significant associations were observed with the Blood derived ranking.

**Table 3:** **Top cell-type enriched gene set enrichment results across three age-associated in the mapped Zeisel dataset**.

			BA11/47				Prefrontal		Blood
Zeisel class	Cell count	Mapped tasic type	Tasic AUROC	Tasic *q*	Zeisel AUROC	*p*	AUROC	*p*	*p*
Oligo2	98	Oligo Opalin	0.73	0.005	0.72	0.0001	0.65	0.0005	0.49
Oligo3	87	Oligo Opalin	0.73	0.005	0.66	0.0009	0.63	0.0048	0.17
Oligo4	110	Oligo Opalin/9630013A20Rik	0.73	0.005	0.66	0.0003	0.62	0.0052	0.18
Oligo5	120	Oligo Opalin	0.73	0.005	0.74	0.0001	0.66	0.0002	0.71
Oligo6	360	Oligo Opalin	0.73	0.005	0.74	0.0001	0.66	0.0001	0.56
S1PyrL23	74	L2/3 Ptgs2	0.46	0.005	0.39	0.0001	0.43	0.0019	0.26
Astro1	68	Astro Aqp4	0.67	0.015	0.7	0.0007	0.74	0.0003	0.72
Astro2	61	Astro Aqp4	0.67	0.015	0.68	0.0019	0.71	0.0016	0.54
CelltypeNonSpecific	NA	CelltypeNonSpecific	0.37	0.02	0.37	0.022	0.31	0.0038	0.57
S1PyrL4	26	L4 Arf5	0.41	0.024	0.44	0.039	0.47	0.12	0.58
S1PyrL5a	28	L5a Batf3	0.46	0.024	0.52	0.62	0.5	0.3	0.65
Oligo1	45	Oligo 9630013A20Rik	0.63	0.048	0.61	0.042	0.62	0.066	0.96

We next evaluated our above findings without the use of mouse datasets to test if using only human data changes our results due to species specific expression signatures between mouse and human cortex (Zeng et al., 2012). We tested the top 21 most specific genes for six cell-types derived from transcriptome profiles of healthy human temporal cortex that were obtained through surgery (Darmanis et al., 2015). Unlike the Tasic and Zeisel datasets, these gene lists were not obtained with our gene selection methods, suggesting that our results were not specific to our thresholded *z*-score technique. While at a coarser resolution of cell identity, these human gene lists confirm our findings for astrocyte and oligodendrocyte enriched genes (Prefrontal and BA11/47 rankings, AUROC > 0.7, Table 4). While this dataset cannot point to a specific neuron type, age-associated down-regulation was seen for genes specific to neurons (Prefrontal and BA11/47 rankings, AUROC < 0.31). Genes enriched in astrocytes, endothelial and microglia cell-types were significantly upregulated in the Blood age association ranking (AUROC > 0.7, corrected *p* < 0.05). Mirroring our results for the non-specific genes, a list of housekeeping human genes obtained from a cross-tissue analysis are down-regulated across age in all three rankings (AUROC: 0.40–0.42, *p* < 0.00001).

**Table 4:** **Enrichment of top human cell-type enriched genes across the age-associated rankings**.

	Ranking		
Name	BA11/47	Prefrontal	Blood
Astrocytes	0.89***	0.84***	0.91*
Endothelial	0.65	0.81***	0.74*
Microglia	0.39	0.56	0.71*
Neuron	0.31*	0.18***	0.52
Oligo	0.97***	0.7*	0.56
OligoPrecusors	0.29**	0.51	0.68

*p < 0.05; **p < 0.005; ***p < 0.0005 (Bonferroni corrected within a ranking/column).

### Gene-Ontology Driven Dissection of Cell-Type Enrichment

To further examine the age-associated gene rankings, we sought to determine if specific biological processes, subcellular locations, or molecular functions were disrupted in a cell-type enriched manner. We used gene set enrichment analysis within the sets of cell-type enriched genes to determine if specific Gene Ontology terms showed strong age associations. For each cell-type gene group, this analysis first subsets the rankings for a specific cell-type enriched list then tests for enrichment of Gene Ontology annotations within that smaller age-associated gene ranking. This can reveal cases where a molecular process in a specific cell-type may change with age, while all other genes enriched in the cell-type are not consistently up- or down-regulated with age.

**Figure 3:**
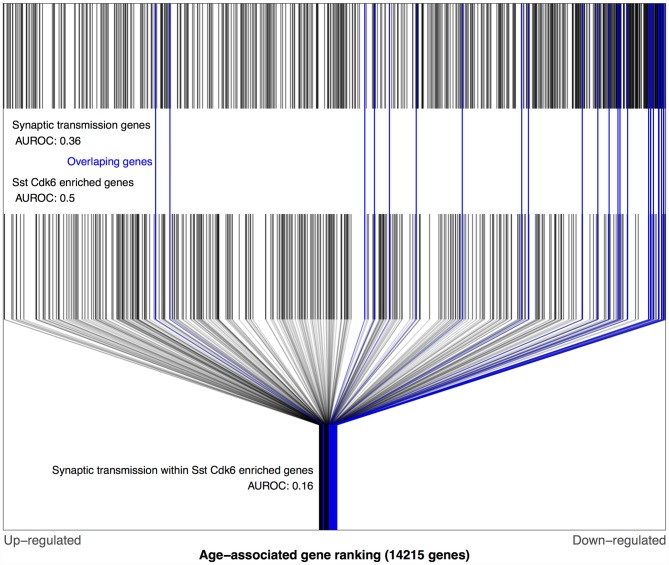
**Distributions of BA11/47 age-related gene rankings in the genomic, somatostatin (Sst) cyclindependent kinase 6 (Cdk6) and synaptic transmission contexts**. Each black line marks a gene, with blue lines for genes enriched in Sst Cdk6 transcriptomic types and annotated for synaptic transmission. The first row of lines shows the overrepresentation of age-associated down-regulation genes within synaptic transmission genes (area under the receiver operating curve), (area under the receiver operating curve, AUROC = 0.36, 660 genes). The second row of marks shows the Sst Cdk6 enriched genes that are evenly distributed across the age-associated gene ranking (AUROC = 0.5, 389 genes). The last row shows only the Sst Cdk6 enriched genes and marks the genes also annotated for synaptic transmission which are mostly age down-regulated (AUROC = 0.16, 25 genes).

Using the Tasic dataset and BA11/47 ranking we computed AUROC values for each of the 38,092 combinations of gene ontology terms and cell type lists. Of these tests, 48 survived multiple test correction (*q* < 0.05), with 45 involving the genes lacking enrichment for a specific cell type. The gene ontology groups involved in these 45 were primarily associated with neurons: synaptic transmission, ion transmembrane transport, axon, sensory perception and behavior (Supplementary Table S1). The highest ranked combination that involved a specific transcriptomic cell-type was the age-associated down-regulation of synaptic transmission genes that were also enriched for a specific somatostatin (Sst) inhibitory cell-type (Sst Cdk6, AUROC = 0.16, *q* < 0.001, Figure 3). All but two of these 25 genes were down-regulated with age (Supplementary Table S2). In comparison, the broader set of Sst Cdk6 enriched genes were randomly distributed across the BA11/47 ranking (AUROC = 0.5). Furthermore, we note that 25 synaptic transmission genes were not more specifically enriched in this cell type in comparison to the whole set of enriched genes (AUROC = 0.57, *p* > 0.5). The cell-cell signaling group was also significant for the Sst Cdk6 cell-type, primarily because the synaptic transmission genes were contained in the group (25 of 36 genes). Cell-cell signaling genes within the vasoactive intestinal peptide (Vip) myosin binding protein C (Mybpc1) cell-type enriched genes provides the only remaining significant age association (18 genes, AUROC = 0.13, *q* < 0.02). All three of these cell-type specific gene ontology group combinations were reproduced when using the Prefrontal age-associated ranking (all *p*-values < 0.0001). In the Blood ranking, the two Sst Cdk6 relationships reverse direction (synaptic transmission: AUROC = 0.69, *p* = 0.04; cell-cell signaling: AUROC = 0.65, *p* < 0.058) and the Vip Mybpc1 finding with cell-cell signaling is not significant (AUC = 0.37, *p* = 0.2).

**Figure 4:**
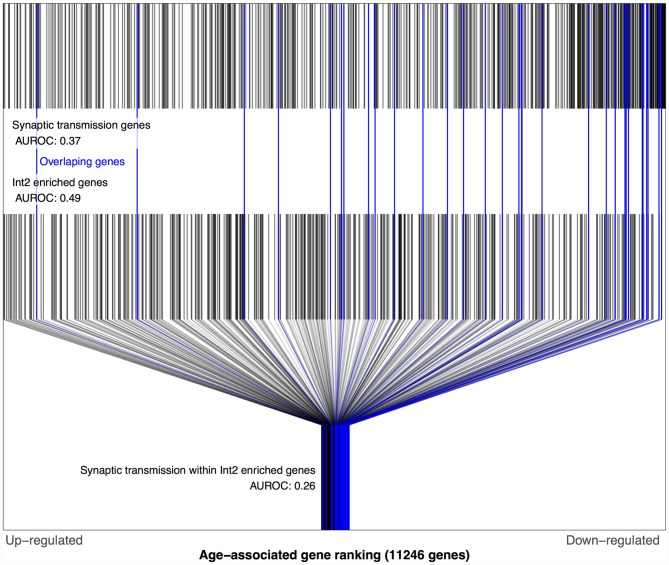
**Distributions of Prefrontal age-related gene rankings in the genomic, synaptic transmission and Int2 (matched to Sst Cdk6) gene set contexts**. Each black line marks a gene, with blue lines for genes enriched in Int2 cells and annotated for synaptic transmission. The first row of lines shows the overrepresentation of age-associated down-regulation genes within synaptic transmission genes (AUROC = 0.37, 591 genes). The second row of marks shows the Int2 enriched genes that are evenly distributed across the age-associated gene ranking (AUROC = 0.49, 467 genes). The last row shows only the Int2 enriched genes and marks the genes also annotated for synaptic transmission which are mostly age down-regulated (AUROC = 0.26, 31 genes).

We next tested the above associations involving Sst Cdk6 and Vip Mybpc1 with the Zeisel dataset. Like above, this required mapping these two transcriptomic cell-types from the Tasic data into the Zeisel subclasses. Of the 16 numbered interneuron subclasses in the Zeisel dataset, Int1 and Int2 have the highest Sst expression. Only the Int2 subclass has cells with non-zero expression for the Cdk6 gene, providing the best match for the Sst Cdk6 transcriptomic class in the Tasic dataset. In the Zeisel dataset, synaptic transmission genes were also age-downregulated in context of the Int2 enriched gene set for both the BA11/47 and Prefrontal age rankings (Figure 4, AUROC < 0.26, *p* < 10^6^) which again reversed direction in the Blood ranking (AUROC = 0.65, *p* < 0.05). Similarly, the Int10 subclass had the highest expression of Vip and Mybpc1 genes across all subclasses in the Zeisel dataset. Corroborating the Tasic results, cell-cell signaling genes are also age-downregulated within the set of Int10 enriched genes for both the BA11/47 and Prefrontal (AUROC < 0.36, *p* < 0.006) but not Blood rankings.

## Discussion

We investigated cell-type enriched genes for age-related changes in three transcriptomic studies of aging. In agreement with past research, we found that genes specifically expressed in oligodendrocytes and astrocytes were up-regulated with age (Erraji-Benchekroun et al., 2005; Primiani et al., 2014; Ianov et al., 2016). This finding is consistent across the two tested sources of neural age-related associations and two cell-type databases and may correspond to changes in the size, metabolism, reactivity, or numbers of neuroglial cells. Detailed studies in monkeys have shown an increase in oligodendrocyte numbers with age (Peters, 2009). For astrocytes, counts in the human brain do not appear to differ across age (Pelvig et al., 2008; Fabricius et al., 2013). Animal studies have found age-dependent increases in astrocytic reactivity and hypertrophy but these findings are considered controversial (Rodríguez-Arellano et al., 2016).

While past studies have observed age-related up-regulation of immune related genes (Erraji-Benchekroun et al., 2005; Cribbs et al., 2012; Primiani et al., 2014; Ianov et al., 2016), we did not observe age-related differences in expression of microglia enriched genes. Most recently, a multiregion expression analysis of the human brain found global age-associated up-regulation of microglia-specific genes (Soreq et al., 2017). In these studies, enrichment of neuroinflammation and microglia associated genes may be due to other factors that affect gene expression in the postmortem brain. These factors include: the region assayed, post-mortem interval and agonal state. Also, conflicting results may be due to a lack of activated microglia in the source cell-type databases that are from younger brains. However, we note that our lack of microglia association agrees with a protein level study that measured translocator protein 18 kDa (TSPO), a marker of activated microglia to assay neuroinflammation. Administration of a TSPO radioligand in healthy subjects and followed by positron emission tomography (PET) found no change across the lifespan (Suridjan et al., 2014). While focused on a single protein, this study was not affected by postmortem factors and supports our finding of stable microglia enriched gene expression.

In terms of direction, we observed broad down-regulation of genes with enriched expression in neurons. However, we observed significant age-related signal for only three of the 42 neuronal transcriptomic types. Genes with enriched expression in layer 2/3 glutamatergic neurons were consistently down-regulated across single-cell datasets and brain based age-associated gene rankings. In support, a study of rhesus monkeys observed a decline of synapses in layers 2/3 (Peters et al., 2008). Studies of neuron vulnerability across layers have found that layer 2/3 neurons were more susceptible to insults in comparison to layers 4 and 5 (Gómez-Isla et al., 1996; Fugistier et al., 2014). In addition, senile plaques were more common in layers 2 and 3 (Duyckaerts et al., 1986). Our results may help link genes to this laminar specific degeneration. Genes enriched in the remaining two neuron types, excitatory neurons in layers 4 and 5a were age-associated in only one of the three dataset-by-ranking combinations.

We speculate that the broad down-regulation of genes with neuron enriched expression, and up-regulation of astrocyte enriched genes are linked. In gray matter, astrocytes are the primary glial cell type. Of the glial types in the Tasic dataset, Astro Aqp4 has the largest cell count. In mice, astrocyte territories and volume increase in old mice (21 months old) when compared to adults (5 months; Grosche et al., 2013). Age up-regulated gene expression and volume increases in astrocytes may signal processes that maintain the same number of tripartite synapses per astrocyte while accommodating reduced neuropil from pyramidal neurons.

We also observed strong down-regulation of genes that were not specific to any cell type. This is consistent across age-related gene rankings and sources of single-cell data. In our gene ontology analysis we found that this non-specific gene set contains many synaptic and ion transport genes which are down-regulated with age in the context of the full non-specific gene set. This may be due to the focus on neurons in the Tasic dataset, combined with broad down-regulation of neuron genes. We note that this downregulation of broadly expressed ion transport genes support the Calcium Hypothesis of Alzheimer’s Disease and Brain Aging which links altered Ca^2+^ homeostasis with brain aging (Khachaturian, 1994; Toescu, 2007). However, this association between non-specific genes and aging holds when using data from other tissues. First, this is the only finding that is reproduced when using the blood derived age-associated gene ranking (using the Tasic but not Zeisel dataset). Second, use of a cross-tissue source of human housekeeping genes that are expressed at constant levels confirms this finding. This strong nonspecific signal may be due to the bulk tissue sources of the age-related rankings. In bulk tissue, nonspecific genes are probably expressed at higher levels, with less noise that could be attributed to differences in cell-type proportions across samples. This stronger signal would provide better estimates of age-associations in comparison to cell-type markers. While we lack a clear understanding of this non-specific signal, our empirical testing procedure was able to control for it in order to accurately highlight transcriptomic cell-types.

Several studies have found that numbers of neurons remain constant while changes at synapses occur with aging (Burke and Barnes, 2006; Loerch et al., 2008; Mostany et al., 2013; Petralia et al., 2014). Our findings support this: genes with enriched expression in the majority of the neuronal transcriptomic types do not show age-related expression patterns. This is coupled with strong age-downregulation of synaptic transmission genes and a broad down-regulation of neuron enriched genes in general. Splitting our cell-type enriched gene lists by gene ontology groups helped focus our findings by marking two inhibitory transcriptomic cell-types with stronger than expected down-regulation of synaptic transmission and cell-cell signaling genes. In contrast, the broader set of genes enriched in these cell-types are not age up- or down-regulated. These findings were robust to different sources of cell-type expression and age-associated rankings. These inhibitory cells represent 14.5% of the Vip and 7.5% of the Sst cells in the Tasic dataset. More broadly, Vip and Sst interneurons have been linked to neuronal vulnerability, neuropsychiatric disorders and dementia (Martel et al., 2012; Lin and Sibille, 2015; Wang et al., 2015; Deng and Jin, 2017). At the synaptic level, we note that Sst and Vip interneurons inhibit each other. In the visual cortex, Sst neurons inhibit all neuron types except other Sst neurons, while Vip neurons preferentially inhibit Sst interneurons (Pfeffer et al., 2013). Our work suggests follow-up studies that examine the age-related changes in the synapse, should target Sst and Vip neurons marked by Cdk6 and Mybpc1, respectively.

There are some limitations of our study. We relied upon mouse single cell expression data for characterizing the human age-related data. Homologous genes and cell-types may not match well across the two species (Zeng et al., 2012), but our use of large enriched gene sets should counter these effects in aggregate. In addition, we employed human single cell expression marker genes to reproduce some findings for cell-type classes that provide the resolution needed (Darmanis et al., 2015). Similarly, while we used only cortical data, the subregions varied from occipital, somatosensory, orbitofrontal and prefrontal cortices. We note that the cerebral cortex is a homogeneous brain region and past studies of age-associated genes have found patterns to be conserved across brain regions (Jaffe et al., 2014). Our limited neuron specific findings may be due to the small sizes of neuronal cell clusters in the single cell data, which had an overrepresentation of neurons. At the same time, these cells would contribute a relatively small portion of the expression signal in the bulk tissue sources for the aging studies. Lastly, another limit is the different classifications of transcriptomic-types across datasets. Going forward, we believe more single cell datasets will coalesce our understanding and classification of cell-types in the brain.

## Conclusion

We found robust age-related up-regulation of genes highly expressed in oligodendrocytes and astrocytes. Genes expressed highly in layer 2/3 glutamatergic neurons were down-regulated across age. Genes not specific to any neural cell type were also down-regulated, possibly due to the bulk tissue source of the age-related genes. Analyses restricted to gene ontology groups highlights strong down-regulation of synaptic transmission and cell-cell signaling genes in the Sst neuron subtype that expresses Cdk6 and in the Vip neuron subtype expressing Mybpc1. These restricted findings provide new insight into which specific cell-type may be susceptible to aging, and suggest age-related synaptic changes in specific inhibitory neuron subtypes. Broadly, our findings suggest that further investigation of oligodendrocyte and astrocyte function across age is needed.

## Ethics Statement

This study reused public data for statistical analysis. That data was already collected under the previous approvals.

## Author Contributions

ES, LF and GCT: conception and design of the work. HO, TM and LF: analysis. LF and ES: drafting and revising the work. All authors approved the manuscript.

## Conflict of Interest Statement

The authors declare that the research was conducted in the absence of any commercial or financial relationships that could be construed as a potential conflict of interest.

## Footnotes

^1^http://casestudies.brain-map.org/celltax/download_archive

^2^https://storage.googleapis.com/linnarsson-lab-www-blobs/blobs/cortex/expression_mRNA_17-Aug-2014.txt

^3^https://github.com/oganm/homologene

## Abbreviations

Astro Aqp4: Astrocyte cells marked by Aqp4
Endo Xdh: Endothelial cells marked by Xdh
Igtp: GABAergic neurons marked by Interferon gamma-induced GTPase
L2 Ngb: Layer 2 glutamatergic neurons marked by Ngb
L2/3 Ptgs2: Layer 2/3 glutamatergic neurons marked by Ptgs2
L4 Arf5: Layer 4 glutamatergic neurons marked by Arf5
L4 Ctxn3: Layer 4 glutamatergic neurons marked by Ctxn3
L4 Scnn1a: Layer 4 glutamatergic neurons marked by Scnn1a
L5 Ucma: Layer 5 glutamatergic neurons marked by Ucma
L5a Batf3: Layer 5a glutamatergic neurons marked by Batf3
L5a Hsd11b1: Layer 5a glutamatergic neurons marked by Hsd11b1
L5a Pde1c: Layer 5a glutamatergic neurons marked by Pde1c
L5a Tcerg1l: Layer 5a glutamatergic neurons marked by Tcerg1l
L5b Cdh13: Layer 5b glutamatergic neurons marked by Cdh13
L5b Chrna6: Layer 5b glutamatergic neurons marked by Chrna6
L5b Tph2: Layer 5b glutamatergic neurons marked by Tph2
L6a Car12: Layer 6a glutamatergic neurons marked by Car12
L6a Mgp: Layer 6a glutamatergic neurons marked by Mgp
L6a Sla: Layer 6a glutamatergic neurons marked by Sla
L6a Syt17: Layer 6a glutamatergic neurons marked by Syt17
L6b Rgs12: Layer 6b glutamatergic neurons marked by Rgs12
L6b Serpinb11: Layer 6b glutamatergic neurons marked by Serpinb11
Micro Ctss: Microglia marked by Ctss
Ndnf Car4: Neurogliaform cells marked by Neuron-derived neurotrophic factor and Car4
Ndnf Cxcl14: Neurogliaform cells marked by Neuron-derived neurotrophic factor and Cxcl14
Oligo 9630013A20Rik: Oligodendrocyte cells marked by 9630013A20Rik
Oligo Opalin: Oligodendrocyte cells marked by Opalin
OPC Pdgfra: Oligodendrocyte progenitor cells cells marked by Pdgfra
Pvalb Cpne5: Parvalbumin GABAergic neurons marked by Cpne5
Pvalb Gpx3: Parvalbumin GABAergic neurons marked by Gpx3
Pvalb Obox3: Parvalbumin GABAergic neurons marked by Obox3
Pvalb Rspo2: Parvalbumin GABAergic neurons marked by Rspo2
Pvalb Tacr3: Parvalbumin GABAergic neurons marked by Tacr3
Pvalb Tpbg: Parvalbumin GABAergic neurons marked by Tpbg
Pvalb Wt1: Parvalbumin GABAergic neurons marked by Wt1
Smad3: GABAergic neurons marked by SMAD family member 3
SMC Myl9: Smooth muscle cells marked by Myl9
Sncg: GABAergic neurons marked by synuclein gamma
Sst Cbln4: Somatostatin GABAergic neurons marked by Cbln4
Sst Cdk6: Somatostatin GABAergic neurons marked by Cdk6
Sst Chodl: Somatostatin GABAergic neurons marked by Chodl
Sst Myh8: Somatostatin GABAergic neurons marked by Myh8
Sst Tacstd2: Somatostatin GABAergic neurons marked by Tacstd2
Sst Th: Somatostatin GABAergic neurons marked by Th
Vip Chat: Vip GABAergic neurons marked by Chat
Vip Gpc3: Vip GABAergic neurons marked by Gpc3
Vip Mybpc1: Vip GABAergic neurons marked by Mybpc1
Vip Parm1: Vip GABAergic neurons marked by Parm1
Vip Sncg: Vip GABAergic neurons marked by Sncg
Zeisel cell types: Astro1: Astrocyte cells, subclass 1
Astro2: Astrocyte cells, subclass 2
Oligo1: Oligodendrocyte cells, subclass 1
Oligo2: Oligodendrocyte cells, subclass 2
Oligo3: Oligodendrocyte cells, subclass 3
Oligo4: Oligodendrocyte cells, subclass 4
Oligo5: Oligodendrocyte cells, subclass 5
Oligo6: Oligodendrocyte cells, subclass 6
S1PyrL23: Pyramidal neurons from layer 2/3 of the somatosensory cortex
S1PyrL4: Pyramidal neurons from layer 4 of the somatosensory cortex
S1PyrL5a: Pyramidal neurons from layer 5a of the somatosensory cortex.
